# Factors associated with assertiveness among Japanese community pharmacists: a cross-sectional study

**DOI:** 10.1186/s40780-025-00410-z

**Published:** 2025-01-23

**Authors:** Mitsuaki Ishii, Sachiko Ozone, Shoichi Masumoto, Tetsuhiro Maeno

**Affiliations:** Department of Primary Care and Medical Education, Institute of Medicine, University of Tsukuba, 1-1-1 Tennodai, Tsukuba, Ibaraki 305–8575 Japan

**Keywords:** Assertive self-expression, Community pharmacists, Interprofessional communication, Medication safety

## Abstract

**Background:**

Community pharmacists play a crucial role in promoting medication safety within the community healthcare team. Effective communication by community pharmacists with other health professionals is essential to facilitate the sharing of patient healthcare information. In the context of information sharing between physicians and community pharmacists, assertive self-expression (defined as ‘a style of openly expressing one's needs and feelings while respecting others’) is beneficial. The aim of this study is to identify factors associated with assertive self-expression among community pharmacists.

**Methods:**

A cross-sectional study was conducted by surveying 3,446 Japanese community pharmacists working at pharmacies across 10 prefectures. Participants were invited to complete a survey form by email and assessed for assertive self-expression using the Interprofessional Assertiveness Scale. Characteristics of participants and pharmacies were compared using univariate analysis. A generalized linear model was used to explore the factors associated with assertive self-expression.

**Results:**

A total of 961 responses by community pharmacists were included in the analysis. Univariate analysis identified significant differences in assertive self-expression scores based on age, employment status, education, years of working experience as a pharmacist, pharmacist home visit service, and participation in joint regional workshops or conferences. After adjustment, participation in joint regional workshops or conferences was significantly associated with assertive self-expression (odds ratio, 1.037; 95% confidence interval, 1.005–1.070; *p* = 0.023).

**Conclusions:**

This study showed that assertive self-expression among community pharmacists was associated with participation in joint regional workshops and conferences. Further research is needed to examine whether enhancing assertive self-expression facilitates pharmacists' interprofessional communication skills and improves medication safety.

## Introduction

Community pharmacists are required to perform their role in the healthcare team to ensure medication safety [1]. As medication safety can be compromised by incorrect administration, inappropriate monitoring, medication errors and communication problems, community pharmacists need to provide adequate medication support [2]. Additionally, in the Japanese healthcare setting, outpatients can freely choose the medical institutions where they receive care. Therefore, the medical information for outpatients is fragmented between institutions, and the importance of sharing medication information between community pharmacists and physicians has been pointed out [3]. Effective communication between community pharmacists and physicians is an important factor in achieving medication safety when sharing patient medication information.

Communication is defined as “the transmission of information, which may be by verbal (oral or written) or nonverbal means” [4]. Healthcare professionals have different communication styles due to differences in training and duties, which can cause mutual stress and poor communication [5]. Pharmacists have been reported to adopt a style that prioritizes the avoidance of confrontation, a style that allows them to impose their own ideas, or a style that focuses on leading others to a mutually acceptable pharmacotherapy; these styles have evolved in a context in which most communication with other professions revolves around pointing out inappropriate pharmacotherapy [6]. Understanding the nature of pharmacists' communication styles is useful to promote better communication with other professions.

Assertiveness is a communication style that is effective for information sharing among healthcare professionals. Assertiveness is defined as “an adaptive style of communication in which individuals express their feelings and needs directly while maintaining respect for others” [7]. There are three types of self-expression associated with assertiveness: nonassertive self-expression, which prioritizes others and downplays one's self; aggressive self-expression, which focuses on imposing one's own opinions; and assertive self-expression, which attempts to enhance mutual understanding while respecting both oneself and others. Assertiveness is a style of self-expression that is assertive rather than nonassertive or aggressive [8]. In the field of patient safety, nurses with high levels of assertiveness have been shown to have higher willingness to speak up about patient safety concerns [9, 10], and it has been reported that assertiveness communication training promotes the willingness to speak up about patient safety [11]. With regard to pharmacists, qualitative research has reported that assertive self-expression by pharmacists when suggesting prescriptions to physicians may influence the implementation of prescribing changes by physicians [12]. We also found that community pharmacists with higher levels of assertive self-expression experienced more frequent pharmacist-initiated prescription changes through medication review [13]. On top of that, the 2022 revision of the Model Core Curriculum for Pharmaceutical Education in Japan highlights the importance of assertive communication in sharing information with other professionals [14].

In order to promote patient safety, it is considered useful to understand the characteristics of healthcare professionals with a high degree of assertiveness and to encourage their practice. Research on nurses has reported that those belonging to the older age groups have lower levels of assertiveness, while those in the psychiatric, educational and administrative departments have higher levels of assertiveness [15]. It has also been reported that a higher level of assertiveness correlates with clarity of role in the workplace [16]. However, to the best of our knowledge, there are no studies investigating the characteristics associated with assertiveness among community pharmacists, although identifying the characteristics of pharmacists with high assertive self-expression attitudes may be valuable in facilitating information sharing for improving medication safety. Therefore, the aim of this study was to identify factors associated with assertive self-expression among community pharmacists.

## Methods

### Research design and participants

The dataset used for this study was obtained from a cross-sectional study in which community pharmacist assertiveness was the explanatory variable and pharmacist-led prescribing change was the outcome variable [13]. The study subjects were 3,446 community pharmacists working at pharmacies part of the X pharmacy chain, with locations in Tokyo and 9 prefectures that were available for the survey.

### Data collection

Participants were invited to participate in this study via their regional managers through an e-mail, which explained the purpose of the study and included a URL and QR code that would allow participants to access the survey form. There was no password protection on the survey form site, and it was explained that responses were to be submitted only once. There were no responses with missing data. The survey was conducted in each region for one month between May and October 2022. Google Forms were used for data collection.

#### Outcome variable

The outcome variable was the assertive self-expression attitude of community pharmacists. We assessed assertive self-expression attitude using the assertive self-expression (AS) domain score of the Interprofessional Assertiveness Scale (IAS) [17]. IAS was designed to assess three self-expression factors related to assertiveness when participating in multi-professional team practice. In our previous study, we found that community pharmacists with higher levels of assertive self-expression experienced more frequent pharmacist-initiated prescription changes through medication review and that neither nonassertive nor aggressive self-expression was associated with such an outcome [13]. Based on these findings, we selected AS as an indicator of assertiveness in the context of medication safety. AS consists of seven items, rated on a six-point Likert scale from 1 (not at all applicable) to 6 (very applicable). Possible scores range from 7 to 42, with higher scores indicating a higher level of assertive self-expressive style.

#### Variables

We identified independent factors associated with communication between physicians and pharmacists through a literature review [18–21]. We included participant characteristics (age, gender, employment status, management or managerial experience, education, number of processed prescriptions per week, working hours per week, years of working experience as a pharmacist, family pharmacist service implementation, pharmacist home visit service implementation, and participation in joint regional workshops or conferences) and pharmacy characteristics (type of institution that provided the largest number of prescriptions per month and distance from the physician to the pharmacy). Participation in joint regional workshops or conferences is an opportunity to enhance communication with other professionals, which has been considered a variable related to collaboration with other professionals in a previous study [20]. Therefore, we assumed that it is related to assertive self-expression, a communication style aimed at enhancing mutual understanding. Although we did not directly measure the frequency of communication between community pharmacists and physicians in this study, we referenced related variables identified in a previous study which identified significant associations between communication frequency and factors such as pharmacists' age and the proximity of healthcare facilities [21]. Thus, we included these factors as confounders to account for contextual influences on communication behaviors. Distance from the physician to the pharmacy was categorized as (i) ≥ 5-min walking distance, (ii) < 5-min walking distance, or (iii) at the same site, based on a previous study related to physician-pharmacist collaboration [21]. 

### Statistical analysis

Descriptive statistics were conducted on participant and pharmacy characteristics. The differences in AS scores were examined using the Mann–Whitney U test and Kruskal–Wallis test with Bonferroni correction based on score distribution. For the relationship between continuous variables, Spearman correlation analysis was used to investigate the correlation between AS score and independent variables.

We investigated the association between AS score and the community pharmacist characteristics using a Generalized Linear Model (GLM) with gamma distribution. To eliminate the possibility of multicollinearity, important explanatory variables were considered based on the association among variables to determine which to include in the multivariable analysis. The following explanatory variables were used: age, gender, employment status, management or managerial experience; these were selected because they have been shown to be associated with assertiveness among healthcare professionals in previous studies [15, 22]. Pharmacists' home visiting services and participation in joint regional workshops or conferences were selected because their median values were significantly different in univariate comparisons (Hosmer–Lemeshow test with a cutoff point set at *p* < 0.25) [23]. Education and years of working experience as a pharmacist were excluded because they were both strongly correlated with age. In this study, the threshold for statistical significance was *p* < 0.05 (two-tailed).

In the multivariable regression analysis, a 15–20 sample for each predictor variable is desirable [24, 25]. In this study, a total of 7 variables or nonreference levels was assumed: each of the 5 continuous and binary variables represents 1 variable, and the 3-level categorical variable of gender represents 2 nonreference variable levels; it was calculated that 140 samples or more were needed to carry out a valid analysis. Statistical analyses were conducted using SPSS Statistics, version 28 (IBM Corp, Armonk, NY, USA).

### Ethical considerations

The study was conducted in accordance with the Declaration of Helsinki, and approved by the ethics committee of the Institute of Medicine, University of Tsukuba (No. 1743). All participants were volunteers and checked the box on the survey form indicating their willingness to participate.

## Results

### Descriptive statistics

**Fig. 1:**
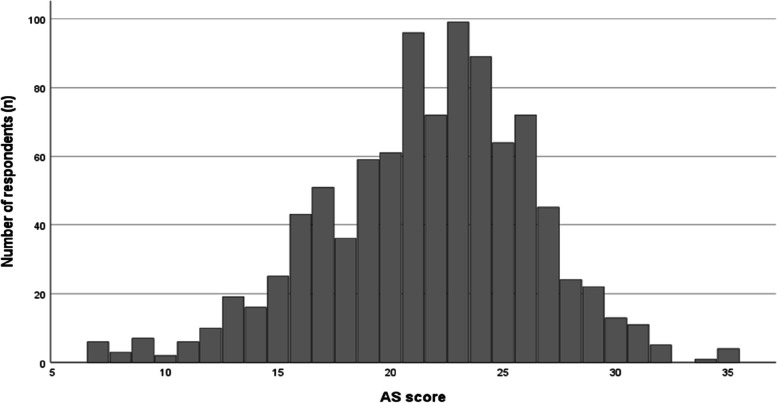
Distribution of AS score responses. AS, assertive self-expression

A total of 1,028 respondents accessed the survey form site. After excluding 59 participants who did not agree to participate, 6 participants who were not working during the hours when they could contact physicians and 2 participants who answered incorrectly, we analyzed the remainder of 961 (27.9%) responses. The mean age (standard deviation) of the respondents was 37.9 (10.8); 531 respondents (55.3%) self-identified as women (Table 1). The median AS score (interquartile range) was 22.0 (19.0–25.0), and the AS scores were non-normally distributed (Shapiro–Wilk test *p* < 0.01) (Fig. 1).

**Table 1:** Participant characteristics (n = 961)

Characteristic	Total
Age, years, median (IQR)	35 (30-45)
Gender, n (%)	
Women	531 (55.3)
Men	396 (41.2)
Prefer not to say	34 (3.5)
Employment status, n (%)	
Full-time	656 (68.3)
Part-time	305 (31.7)
Management or managerial experience, n (%)	
Yes	558 (58.1)
No	403 (41.9)
Education, n (%)	
Bachelor degree (4 years)	399 (41.5)
Bachelor degree (6 years)	514 (53.5)
Master's or doctorate degree	48 (5.0)
Number of processed prescriptions per week, n (%)	
< 50	171 (17.8)
51–100	439 (45.7)
101–150	251 (26.1)
≥ 151	100 (10.4)
Working hours per week, median (IQR)	40 (32-40)
Years of working experience as a pharmacist, median (IQR)	8 (4-15)
Family pharmacist service, n (%)	
Yes	200 (20.8)
No	761 (79.2)
Pharmacist home visit service, n (%)	
Yes	284 (29.6)
No	677 (70.4)
Participation in joint regional workshops or conferences, n (%)	
Yes	353 (36.7)
No	608 (63.3)
Area, n (%)	
Tokyo	108 (11.2)
Kanagawa	364 (37.9)
Saitama	140 (14.6)
Chiba	244 (25.4)
Six other prefectures (Fukui, Ishikawa, Nagano, Niigata, Toyama, Yamanashi)	105 (10.9)
Type of institutions providing the most prescriptions, n (%)	
Clinic	574 (59.7)
Hospital	387 (40.3)
Distance from the physician to the pharmacy, n (%)	
More than a 5-min walking distance	500 (52.0)
Less than a 5-min walking distance	368 (38.3)
At the same site	93 (9.7)
Total AS score, median (IQR)	22.0 (19.0–25.0)

*IQR* interquartile range

### Univariate and multivariable analyses

Variables that significantly differed at the level of significance (*p* < 0.25) set as the level for selecting variables to be used in the multivariable analysis were: age, employment status, education, years of working experience as a pharmacist, pharmacist home visit service, and participation in joint regional workshops or conferences (Table 2). In a multivariable analysis using GLM, we found a significant positive association between AS score and participation in joint regional workshops or conferences (OR, 1.037; 95% CI, 1.005–1.070; *p* = 0.023) and no significant association with age, employment status or pharmacist home visit service (Table 3).

**Table 2:** Participant characteristics by assertive self-expression score (n = 961)

Variable	AS score median (IQR)	*p* value	Spearman correlation coefficient
Age, years, median (IQR)^a^	35.0 (30.0–45.0)	< 0.001	-0.132
Gender			
Women	22.0 (19.0–25.0)	0.475	
Men	22.0 (19.0–25.0)		
Prefer not to say	20.5 (17.0–25.0)		
Employment status			
Full-time	21.0 (18.0–24.0)	0.004	
Part-time	22.0 (19.0–25.0)		
Management or managerial experience			
Yes	22.0 (19.0–25.0)	0.775	
No	22.0 (19.0–25.0)		
Education			
Bachelor degree (4 years)	21.0 (18.0–24.0)	< 0.001	
Bachelor degree (6 years)	23.0 (19.0–25.09		
Master's or doctorate degree	22.0 (20.0–25.0)		
Number of processed prescriptions per week			
< 50	22.0 (19.0–25.0)	0.682	
51–100	22.0 (19.0–25.0)		
101–150	22.0 (18.0–25.0)		
≥ 151	23.0 (19.0–25.0)		
Working hours per week, median^a^	40.0 (32.0–40.0)	0.417	0.026
Years of working experience as a pharmacist, median^a^	8.0 (4.0–15.0)	< 0.001	-0.119
Family pharmacist service			
Yes	22.0 (19.0–25.0)	0.398	
No	22.0 (19.0–25.0)		
Pharmacist home visit service			
Yes	23.0 (19.0–25.0)	0.037	
No	22.0 (19.0–25.0)		
Participation in joint regional workshops or conferences			
Yes	23.0 (19.0–25.0)	0.041	
No	22.0 (19.0–24.0)		
Area			
Tokyo	22.0 (18.0–24.0)	0.274	
Kanagawa	23.0 (19.0–25.0)		
Saitama	22.0 (19.0–24.0)		
Chiba	21.0 (19.0–25.0)		
Six other prefectures (Fukui, Ishikawa, Nagano, Niigata, Toyama, Yamanashi)	22.0 (19.0–25.0)		
Type of institutions providing the most prescriptions			
Clinic	22.0 (19.0–25.0)	0.705	
Hospital	22.0 (19.0–25.0)		
Distance from the physician to the pharmacy			
More than a 5-min walking distance	22.0 (19.0–25.0)	0.256	
Less than a 5-min walking distance	22.0 (19.0–25.0)		
At the same site	23.0 (19.0–26.0)		

Medians were compared using the Mann–Whitney U test or Kruskal–Wallis test

*AS* assertive self-expression, *IQR* interquartile range

^*^Univariate analysis of the association with AS score (Spearman's correlation coefficient)

**Table 3:** Association of AS score with characteristics of community pharmacists

Variable	Odds ratio	95% confidence interval	*p*
Age, years	0.999	0.997-1.000	0.079
Gender			
Women	Reference		
Men	0.972	0.938-1.007	0.121
Prefer not to say	0.950	0.876-1.030	0.221
Employment status			
Part-time	Reference		
Full-time	1.040	0.995-1.087	0.084
Management or managerial experience			
No	Reference		
Yes	0.990	0.957-1.025	0.578
Pharmacist home visit service			
No	Reference		
Yes	1.013	0.979-1.049	0.444
Participation in joint regional workshops or conferences			
No	Reference		
Yes	1.037	1.005-1.070	0.023

*AS* assertive self-expression

## Discussion

The present study was an exploratory examination of factors associated with assertive self-expression among community pharmacists. The results showed that participation in joint regional workshops and conferences was associated with assertive self-expression. To the best of our knowledge, little evidence exists on assertive self-expression among community pharmacists, and we believe that our findings provide valuable insights into understanding assertive self-expression for this population of healthcare professionals.

Our study revealed that community pharmacists’ participation in joint regional workshops or conferences was positively associated with assertive self-expression. Joint regional workshops or conferences are intended to facilitate better communication between professionals; pharmacists have been reported to show a willingness to participate in such events in order to better understand the role of other professionals. [26, 27] Assertive self-expression is a style of self-expression that enhances mutual understanding and attempts to consider the points of view expressed by others [8]. Although the present study found a positive association between the experience of participating in joint regional workshops or conferences and assertive self-expression among community pharmacists, the causal relationship between these variables is unclear and further research is needed.

Our results showed that age and management or managerial experience, which were significantly different in the univariate analysis, were no longer associated with assertive self-expression in the multivariable analysis. With regards to age, studies of nurses and midwives have shown that those in younger age groups exhibit higher levels of assertiveness [15]. On the other hand, no association between age and assertiveness has been reported among physicians [28]. In community pharmacists, similar to physicians, no association was found between assertive self-expression and age. Regarding management or managerial experience, assertive communication by leaders in an organization has been reported to be associated with better relationships among employees and with behaviors by employees that improve organizational functioning [29]. Research in nursing contexts has reported that nurses in managerial positions display a higher level of assertiveness than those who are in more subordinate roles [30]. Our results indicate that factors affecting assertiveness vary depending on the healthcare professional and their role, as no association was found among community pharmacists.

Our study had some limitations. First, our study was conducted among pharmacists working in pharmacies which are attached to larger drugstores; approximately 10% of community pharmacies that accept prescriptions have been reported to be attached to a drugstore [31]. Our results should be considered with caution in generalizing to community pharmacists in Japan. We believe that further research including community pharmacists working in pharmacies that mainly process prescriptions is needed to make the study results more generalizable. Second, there is a possibility of social desirability bias due to participation by community pharmacists interested in showing assertiveness by responding to the self-administered survey. It is possible that this might have skewed the association between assertive self-expression attitudes and other factors. Finally, although we conducted our analysis using multivariable analysis, we may not have collected all potential factors. There may also be unmeasured confounding factors, which may introduce bias in the interpretation of the results.

## Conclusions

Participation in joint regional workshops or conferences is associated with assertive self-expression among community pharmacists. These findings may be useful in facilitating assertive self-expression among community pharmacists and thus lead to better information sharing with other health professionals. To verify the causal relationship suggested by our findings, we believe that it is necessary to examine whether repeated participation in joint regional workshops or conferences improves assertive self-expression among community pharmacists. Further research is needed to examine whether enhancing assertive self-expression facilitates pharmacists' interprofessional communication and improves medication safety.

## Data Availability

The datasets used and analyzed during the current study are not publicly available because they contain information that could compromise the privacy of research participants, but de-identified datasets are available from the corresponding author on reasonable request.

## Abbreviations

AS: Assertive self-expression
GLM: Generalized Linear Model
IAS: Interprofessional Assertiveness Scale
